# Transcriptomics Revealed Differentially Expressed Transcription Factors and MicroRNAs in Human Diabetic Foot Ulcers

**DOI:** 10.3390/proteomes12040032

**Published:** 2024-11-05

**Authors:** Vikrant Rai

**Affiliations:** Department of Translational Research, Western University of Health Sciences, 309 E. Second Street, Pomona, CA 91766-1854, USA; vrai@westernu.edu; Tel.: +1-909-469-7043

**Keywords:** diabetic foot ulcers, non-healing ulcers, amputation, transcription factors, microRNA, therapeutic target

## Abstract

Non-healing diabetic foot ulcers (DFUs) not only significantly increase morbidity and mortality but also cost a lot and drain healthcare resources. Persistent inflammation, decreased angiogenesis, and altered extracellular matrix remodeling contribute to delayed healing or non-healing. Recent studies suggest an increasing trend of DFUs in diabetes patients, and non-healing DFYs increase the incidence of amputation. Despite the current treatment with offloading, dressing, antibiotics use, and oxygen therapy, the risk of amputation persists. Thus, there is a need to understand the molecular and cellular factors regulating healing in DFUs. The ongoing research based on proteomics and transcriptomics has predicted multiple potential targets, but there is no definitive therapy to enhance healing in chronic DFUs. Increased or decreased expression of various proteins encoded by genes, whose expression transcriptionally and post-transcriptionally is regulated by transcription factors (TFs) and microRNAs (miRs), regulates DFU healing. For this study, RNA sequencing was conducted on 20 DFU samples of ulcer tissue and non-ulcerated nearby healthy tissues. The IPA analysis revealed various activated and inhibited transcription factors and microRNAs. Further network analysis revealed interactions between the TFs and miRs and the molecular targets of these TFs and miRs. The analysis revealed 30 differentially expressed transcription factors (21 activated and 9 inhibited), two translational regulators (RPSA and EIF4G2), and seven miRs, including mir-486, mir-324, mir-23, mir-186, mir-210, mir-199, and mir-338 in upstream regulators (*p* < 0.05), while causal network analysis (*p* < 0.05) revealed 28 differentially expressed TFs (19 activated and 9 inhibited), two translational regulators (RPSA and EIF4G2), and five miRs including mir-155, mir-486, mir-324, mir-210, and mir-1225. The protein–protein interaction analysis revealed the interaction of various novel proteins with the proteins involved in regulating DFU pathogenesis and healing. The results of this study highlight many activated and inhibited novel TFs and miRs not reported in the literature so far, as well as the targeted molecules. Since proteins are the functional units during biological processes, alteration of gene expression may result in different proteoforms and protein species, making the wound microenvironment a complex protein interaction (proteome complexity). Thus, investigating the effects of these TFs and miRs on protein expression using proteomics and combining these results with transcriptomics will help advance research on DFU healing and delineate potential therapeutic strategies.

## 1. Introduction

Skin, the protective barrier against environmental pathogens, is involved in the proper fluid balance of the skin by preventing fluid loss. Thus, proper wound healing, efficiently and rapidly, is important for the proper functioning of the skin, and delayed wound healing may result in increased invasion of pathogens [1]. Chronic inflammation, decreased angiogenesis, and altered extracellular matrix (ECM) remodeling contribute to delayed wound healing and chronic ulceration. Various molecular and cellular factors, including damage-associated molecular patterns (DAMPs), toll-like receptors, inflammatory cytokines including interleukin (IL)-1, IL-6, IL-8, and tumor necrosis factor (TNF)-α, immune cells including macrophages, neutrophils, fibroblasts, and keratinocytes, and the interaction between these factors play a critical role in delaying wound healing [2,3,4,5,6]. The four phases of wound healing, including hemostasis, inflammation, resolution, and remodeling, are regulated by various factors, and a change in the expression of these factors may alter wound healing; thus, it is important to delineate the factors regulating the expression of various genes involved in the pathogenesis of wound healing and delayed healing. Transcription factors and microRNAs regulate gene expression coding for proteins at the transcriptional and post-transcriptional levels, respectively [7,8].

Transcription factors (TFs), by binding to regulatory regions, regulate the expression of various genes involved in various pathways involved in wound healing, and the expression of TFs changes over time during wound healing. Keratinocytes originating from stem cells found in the hair follicle and TFs Kruppel-like factor 5 (KLF5), SRY-box transcription factor 9 (SOX9), activator protein 1 (AP1), signal transducer and activator of transcription 3 (STAT3), and GATA-binding factor 6 (GATA6) play a regulatory role in plasticity and differential expression of these cells and immune regulation during wound healing by regulating expression of various genes involved in granulation tissue formation, ECM remodeling, and angiogenesis [1]. TF nuclear factor-erythroid 2-related factor 2 (NRF2) plays a critical role in response to cellular stress (oxidative stress) and also plays a role in wound healing, evidenced by increased *Nrf2* gene expression after full-thickness wounding and decreasing with time [9]. NRF2 plays a role in wound inflammation, immune cell response and profile, regulation of oxidative stress, and phenotypic change in keratinocytes and fibroblasts, however, activation and knocking out of NRF2 have differential effects on various molecular and cellular events during wound healing [9]. Another TF, Foxn1, regulates cutaneous wound healing by promoting epithelial–mesenchymal transition, playing a critical role in wound healing by promoting keratinocyte migration towards the dermis, as evidenced by increased levels of Snail1 and MMP-9 expression and through vimentin/E-cadherin-positive cells in dermal tissue [10]. Cell renewal is promoted by TFs SOX2 and SOX9, while the microvascular environment is supported by SOX7 during cutaneous wound healing [11]. The FOXO family of TFs plays a role in wound healing; however, their effect on wound healing is different in controlling wound healing compared to healing wounds in diabetes. FOXO1 and FOXO3 promote keratinocyte migration via TGF-β in normal wound healing, while the genes affecting the re-epithelialization and keratinocyte migration are negatively regulated in cases of diabetic wound healing [12]. Increased expression and activity of FOXO1 during diabetes induces apoptosis of pericytes and endothelial cells and increases secretion of pro-inflammatory cytokines. Increased FOXO1 expression increases TGF-β in control skin while decreasing in diabetic skin, resulting in increased migration of keratinocytes in control skin, while decreased migration in diabetic skin contributes to delayed wound healing [13].

Immune cells, mainly monocytes and macrophages, regulate the inflammatory response in various phases of wound healing and, by interacting with fibroblasts, keratinocytes, vascular smooth muscle cells, and endothelial cells, directly or via paracrine effect, play a critical role in wound healing [3,4,5,14,15,16]. A study by Zandighar et al. reported 19 TFs, mainly CEBPD, PRDM1, STAT1, IRF7, JUNB, STAT2, NR4A1 (NUR77), STAT4, IKZF, MAFB, KLF4, MITF, EGR2, and IRF1 in regulating heterogeneity of monocytes and macrophages during various phases of wound healing [17]. Parella et al. reported that the administration of TF HoxA3 in diabetic mice promotes wound healing in the presence of hyperglycemia and with aging [18]. Forkhead box M1 (FOXM1) TF has been shown to promote wound healing in diabetic foot ulcers by enhanced M2 polarization involving SEMA3C/NRP2/Hedgehog signaling [19]. Further, inhibition of the cytosolic function of CXXC5 has been associated with enhanced wound healing by promoting angiogenesis and ECM remodeling in diabetic rats [20]. Another study by Zhang et al. reported the role of DNA binding with one-finger (DOF) transcription factors, namely HIGH CAMBIAL ACTIVITY2 (HCA2), TARGET OF MONOPTEROS6 (TMO6), DOF2.1, and DOF6 in healing and regeneration in plants [21].

Fibroblast differentiation and heterogeneity play a critical role in wound healing [5] and TFs and microRNAs (miRs) play a critical role in regulating fibroblast reprogramming during wound healing [22,23]. miR-21 regulates angiogenesis, fibroblast function, keratinocyte proliferation and migration, and inflammatory response [24], and miR-221-3p inhibits the inflammatory response of keratinocytes in promoting wound healing [25]. Further, the regulatory role of various miRs in regulating immune cells, inflammatory response, inflammatory pathways, complement activation, coagulation, oxidative stress, and other signaling involved in wound healing has been documented. Furthermore, time-dependent activation or inhibition of miRs during wound healing has been reported [26,27,28]. These studies suggest that TFs and miRs play a critical role in regulating various molecular and cellular processes during wound healing, and their expression changes during various phases of wound healing. Based on this, the aim of this study was to delineate the TFs and miRs differentially expressed in chronic diabetic foot ulcers compared to the nearby non-ulcerated skin in human patients. The results of this study revealed differentially expressed TFs and miRs and an association between TFs and miRs in ulcerated tissues compared to non-ulcerated tissues. These findings are important in designing novel therapeutics by targeting various proteins whose expression is regulated by differentially expressed TFs and miRs.

## 2. Materials and Methods

Fifty-one samples, both ulcerated and nearby non-ulcerated, were collected from forty-nine DFU with osteomyelitis patients undergoing transmetatarsal (TMA) amputation at Riverside University Hospital System (RUHS) for RNA sequencing. The approval for the study was obtained from IRB, with approval number 1787616-2 from the Institutional Board of Riverside University Hospital System (RUHS) at Moreno Valley, CA, USA, and Western University of Health Sciences at Pomona, CA. All patients above the age of 18 years with DFUs and admitted for amputation were included, while patients with a history of smoking, myocardial ischemia, angina, macroalbuminuria, or any other serious illness were excluded. Informed consent was obtained from the patients for TMA surgery; however, it was waived for this study as the tissues were collected anonymously by the surgeon and staff nurse not involved in the study. The Institutional Review Board approved the study with a waiver for informed consent. The tissues were collected from the ulcerated area of the amputated part, and nearby normal tissue (dermal tissue) was collected from the flap created, as shown in [4]. The patients’ demographics and characteristics with medical history have been previously described in detail [4] and the tissues from the same approved study were used for RNA sequencing.

The samples collected for RNA later at RUHS were transported to Westen University of Health Sciences at Pomona, CA, USA at 4 °C and immediately stored at −80 °C. Out of 50 samples, 20 tissue samples (10 ulcerated and 10 non-ulcerated nearby normal tissues) [4] were used for RNA sequencing and data analysis at the University of California at Los Angeles (UCLA) Technology Center for Genomics & Bioinformatics Department of Pathology & Laboratory Medicine following the methodology discussed in our previous publication [29]. RNA sequencing was conducted after a quality check of the isolated RNA. RNA samples with RIN > 6 were used for sequencing. Further analysis was conducted at the Bioinformatics and Systems Biology Core, Dept. of Genetics, Cell Biology & Anatomy, University of Nebraska Medical Center, Omaha, NE. Ingenuity Pathway Analysis (IPA) (QIAGEN Inc., Germantown, MD, USA, https://digitalinsights.qiagen.com/products-overview/discovery-insights-portfolio/analysis-and-visualization/qiagen-ipa/ (accessed on 19 March 2024)) was conducted for pathway, functional, and network analyses mapped to human and mouse pathway databases. The algorithms developed for use in IPA were followed, as discussed by Krämer et al. [30]. IPA results were uploaded for the D vs. C comparison gene list (*p*-value ≤ 0.05). The pathway, functional, and network analyses mapping to human and mouse pathway databases were generated through the use of IPA (QIAGEN Inc.). The upstream regulators and causal pathway analysis revealed multiple TFs and miRs (activated or inhibited) associated with differentially expressed genes of genes and target molecules in the dataset. Further, network analysis using Networkanalyst.ca was performed to delineate the interactions between TFs and miRs, as well as the genes regulated by them. Transcriptional Regulatory Relationships Unravelled by Sentence-based Text-mining (TRRUST), a curated database of human transcriptional regulatory networks, and the Encyclopedia of DNA Elements (ENCODE), transcription factor and gene target data derived from the ENCODE ChIP-seq data, were used to evaluate the gene–TF interactions, while miRTarBase v9.0 was used for TF-miRNA coregulatory interactions, the literature-curated regulatory interaction information collected from the RegNetwork repository.

## 3. Results

Differentially expressed genes in ulcer tissue compared to control: RNA seq analysis revealed a total of 28,315 genes, out of which 2130 genes were differentially expressed (*p* < 0.05). Out of these 2130 genes, 1102 were upregulated (Log2 fold > 1.5), and 624 were downregulated (negative log2 fold). Of these 624 genes, 375 genes were downregulated with log2Folds < −1.5.

Ingenuity Pathway Analysis (IPA) results revealed activated and inhibited TFs and miRs in ulcerated tissues compared to non-ulcerated tissues from patients with DFUs. IPA revealed 102 transcription factors (21 activated and 9 inhibited), two translational regulators including RPSA and EIF4G2 (one inhibited, RPSA, z-score −2), and seven miRs including mir-486, mir-324, mir-23, mir-186, mir-210, mir-199, and mir-338 (one inhibited, mir-486, z-score −2) in upstream regulators (*p* < 0.05) analysis. The Causal network analysis (*p* < 0.05) revealed 49 TFs (19 activated and 9 inhibited), two translational regulators namely RPSA and EIF4G2 (one inhibited RPSA, z-score −2.191), and five miRs including mir-155, mir-486, mir-324, mir-210, and mir-1225 (one activated; mir-155 with z-score 3.703 and one inhibited; mir-486 with z-score −2) (Table 1).

**Figure 1:**
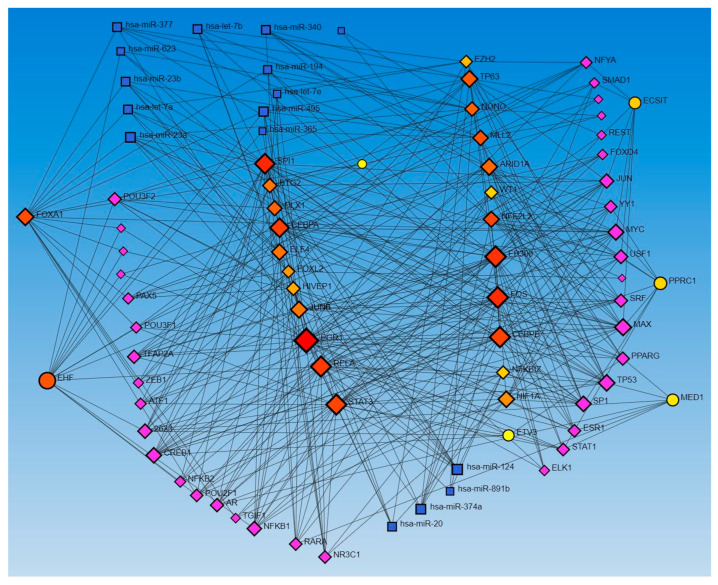
TF-miRNA coregulatory interactions between activated and inhibited TFs and miRs in upstream regulators (IPA analysis): The literature-curated regulatory interaction information was collected from the RegNetwork repository using miRTarBase v9.0 (Inbuilt in Networkanalyst.ca). Blue squares—miRs, pink squares—newly appeared TFs, red/orange and yellow squares and circles—input TFs.

Network analysis with activated and inhibited TFs and miR in upstream regulators and causal networks revealed an association with each other. Network analysis using Networkanalyst.ca for TF-miRNA coregulatory interactions revealed an interaction between activated and inhibited TFs, including ETS homologous factor (EHF), Non-POU domain-containing octamer-binding protein (NONO), TP63, NFKB inhibitor zeta (NFKBIZ), RELA, PPARG related coactivator 1 (PPRC1), forkhead box L2 (FOXL2), FOS, ECSIT Signaling Integrator (ECSIT), JUNB, CCAAT Enhancer Binding Protein Alpha (CEBPA), hypoxia-inducible factor 1 alpha (HIF1A), enhancer of zeste homolog 2 (EZH2), CCAAT Enhancer Binding Protein Beta (CEBPB), STAT3, SPI1 (an ETS-domain transcription factor), E1A-associated protein p300 (EP300), E74-like ETS transcription factor 4 (ELF4), earlt growth response (EGR)1, NFE2 like bZIP transcription factor 2 (NFE2L2), Mediator of RNA polymerase II transcription subunit 1 (MED1), Lysine Methyltransferase 2D (KMT2D), AT-rich interaction domain 1A (ARID1A), HIVEP zinc finger 1 (HIVEP1), Homeobox protein NANOG, forkhead box A1 (FOXA1), Distal-Less Homeobox 1 (DLX1), ETS Variant Transcription Factor 3 (ETV3), BTG anti-proliferation factor 2 (BTG2), Wilms’ tumor 1 (WT1), and catenin beta 1 (CTNNB1) (as input list), with miR-23a and miR-23b (Figure 1). The other miRs, including mir-486, mir-324, mir-186, mir-210, mir-199, and mir-338, did not appear in the limited network analysis. We did the limited network analysis because the first-order interaction analysis showed more than 2000 interactions, and the remaining miRs may be a part of these interactions (limitation of the analysis tool). The analysis also revealed many other TFs and miRs interacting with each other (Figure 1). 

**Figure 2:**
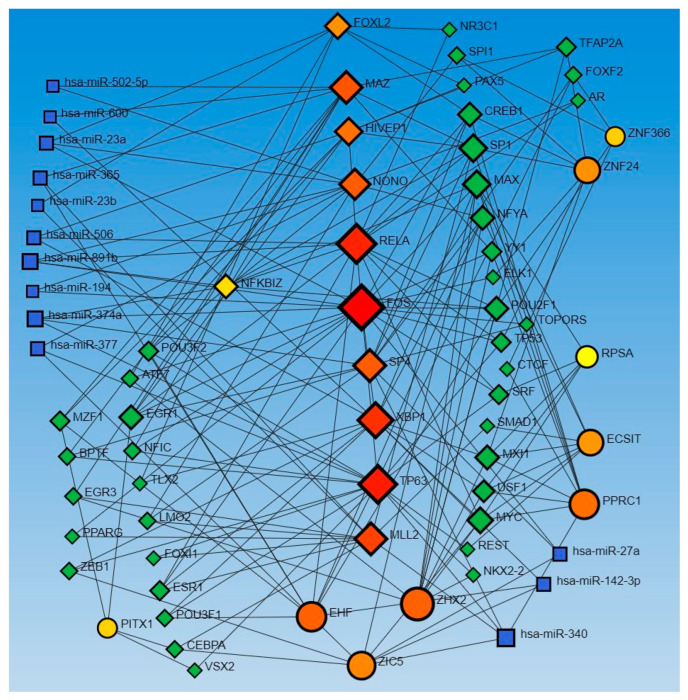
TF-miRNA coregulatory interactions between activated and inhibited TFs and miRs in the causal network (IPA analysis): The literature-curated regulatory interaction information was collected from the RegNetwork repository using miRTarBase v9.0 (Inbuilt in Networkanalyst.ca). Blue squares—miRs, pink squares—newly appeared TFs, red/orange and yellow squares and circles—input TFs.

Similarly, the network analysis with an input list of activated and inhibited TFs from causal network (IPA analysis), including MAZ, NONO, ZIC5, TP63, EHF, EHF, RELA, ECSIT, NFKBIZ, PPRC1, RELA, FOXL2, FOS, FOS, ECSIT, SP4, ZHX2, XBP1, KMT2D, NFKBIZ, ZNF366, KMT2D, HIVEP1, ZNF24, PITX1, HIVEP1, and RPSA, revealed an interaction between TFs and miR-23a and miR-23b. The analysis did not reveal any interaction between the miRs that appeared in the causal network analysis (mir-155, -486, -324, -210, and 1225) but revealed other miRs (blue squares, Figure 2). miR-23a and miR23b were common in both upstream regulators and causal network analysis and appeared in network analyses interacting with various TFs. Network analysis using the causal network TFs input list showed more than 2000 interactions, so we did the limited network analysis, and the remaining miRs appearing in the causal network during IPA analysis may be a part of these 2000 interactions (limitation of the analysis tool).

The network analysis for TF-miRs interaction, using upstream regulators and causal network TFs, revealed miR-340, -377, -374a, -194, -891b, and -365 in both analysis, miR-142-3p, 27a, -506, -600, and -502-5p in causal network analysis alone, and miR-124, -623, -495, let-7a, let-7b, and let-7e in upstream regulators only (Figure 1 and Figure 2).

**Figure 3:**
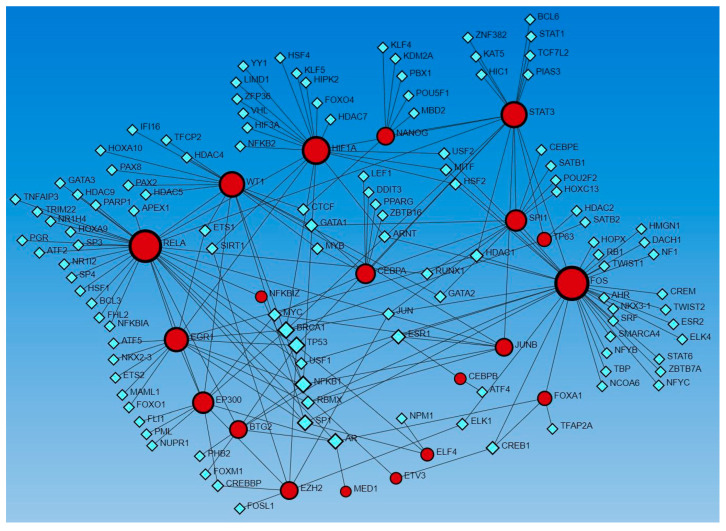
Gene–transcription factors interaction network analysis using the Transcriptional Regulatory Relationships Unraveled by Sentence-based Text-mining (TRRUST) database of human transcriptional regulatory networks. Red circles—input genes (regulated by TFs in upstream regulator analysis), blue squares—TFs regulating these genes.

**Figure 4:**
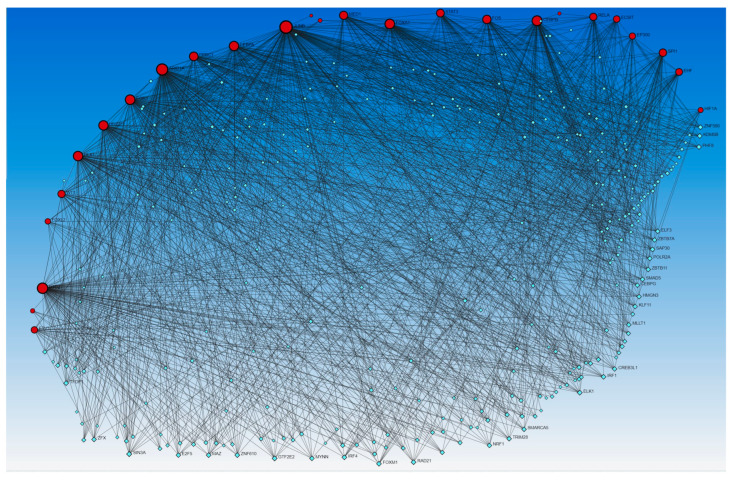
Gene–transcription factors interaction network analysis using the Encyclopedia of DNA Elements (ENCODE) database. Red circles—input genes (regulated by TFs in upstream regulator analysis), blue squares—TFs regulating these genes.

Network analysis revealed the interaction of genes regulated by activated and inhibited TFs with other TFs. Gene expression coding for functional proteins is regulated by TFs, so to delineate the interaction of genes coded by TFs that appeared in upstream regulators, we used Networkanalyst.ca. The analysis revealed interactions between these genes as well as with other TFs using the TRRUST database (Figure 3) and ENCODE database (Figure 4).

**Figure 5:**
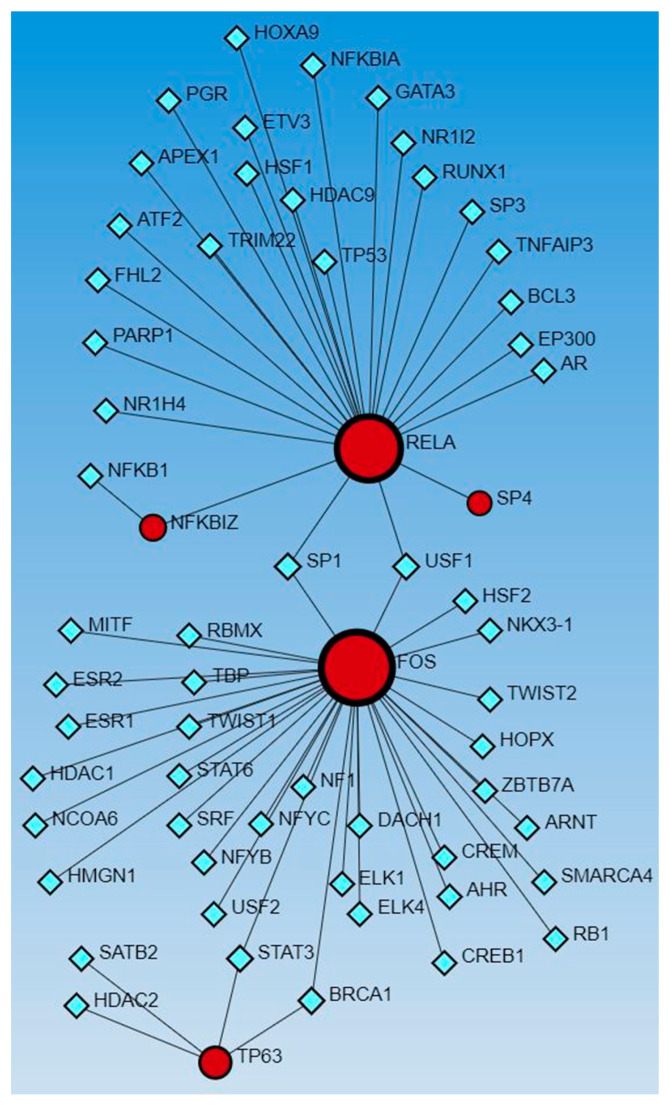
Gene–transcription factors interaction network analysis using Transcriptional Regulatory Relationships Unraveled by Sentence-based Text-mining (TRRUST) database of human transcriptional regulatory networks. Red circles—input genes (regulated by TFs in causal network analysis), blue squares—TFs regulating these genes.

**Figure 6:**
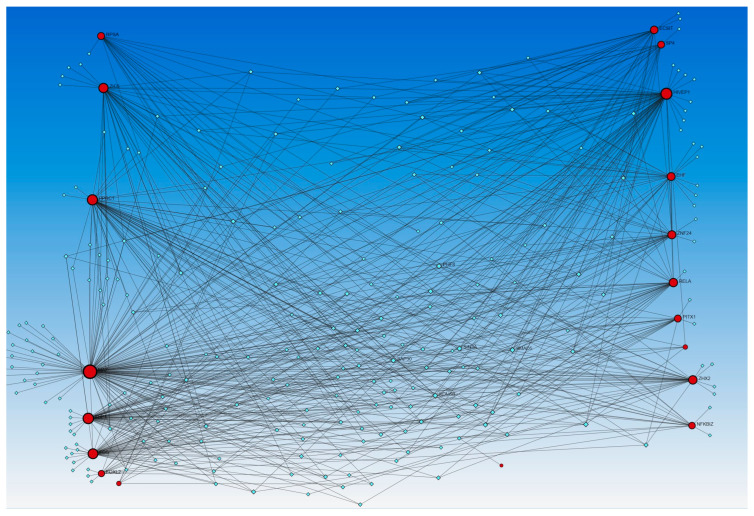
Gene–transcription factors interaction network analysis using the Encyclopedia of DNA Elements (ENCODE) database. Red circles—input genes (regulated by TFs in causal network analysis), blue squares—TFs regulating these genes.

Next, to delineate the interactions between genes coded by activated and inhibited TFs that appeared in the causal network analysis (IPA analysis), Networkanalyst.ca was used. The results revealed the interaction between genes coded by activated and inhibited TFs with other TFs using the TRRUST (Figure 5) and ENCODE (Figure 6) databases. 

TFs including ECSIT, EHF, FOS, FOXL2, NONO, NFKBIZ, PPRC1, RELA, TP63 (all activated), and HIVEP1 and KMT2D (both inhibited) appeared in both upstream regulators and causal network analysis. Upstream regulators showed activated TFs, including CEBPA, CEBPB, EGR1, ELF4, EP300, EZH2, HIF1A, JUNB, MED1, NFE2L2, SPI1, STAT3, and inhibited TFs, including ARID1A, BTG2, DLX1, ETV3, FOXA1, NANOG, and WT1. The causal network analysis revealed activated TFs, including MAZ, SP4, ZIC5, and ZHX2, and inhibited TFs, including KMT2D, PITX1, XBP1, ZNF366, and ZNF24.

## 4. Discussion

TF-miRNA coregulatory interactions revealed miR-23a and miR-23b interacting with various TFs activated or inhibited in tissue samples of ulcerated DFUs compared to non-ulcerated nearby tissues. This suggests a role of miR-23 in regulating gene expression involved in DFU healing during translation and transcription, and the finding is supported by the decreased expression of miR-23a and miR-23b in DFU tissues with increased miR-23c expression [31]. miR-23 appeared in upstream regulation but was neither activated nor inhibited in our data. This may be because the analyzed data showed miR-23 and not miR-23a, 23b, and 23c separately. The appearance of miR-23 in our data suggests that it may regulate wound healing by attenuating angiogenesis (miR-23c) via targeting stromal cell-derived factor-1α (SDF-1α/CXCL12) [31]. However, the role of miR-23a and miR23b during DFU healing needs to be investigated. miR-23a was also found to be involved in regulating angiogenesis during wound healing in rats involving IRF-1 [32]. miR-23a and miR23b are associated with diabetic neuropathy, an important risk factor and contributor to non-healing DFUs [33]. miR-23b promotes keratinocyte migration by attenuating tissue inhibitors of matrix metalloproteinase 3 (TIMP3) expression [34].

miR-27a in the network analysis of causal network TF-miR interaction is expressed on endothelial cells along with its members miR27b and miR-27a/b and is involved in vasculogenesis in Zebrafish. miR-27 upregulates VEGFR activation by inhibiting Sem6A and Sprouty 2 intracellularly and downregulates the antiangiogenic marker thrombospondin (TSP)-I to promote angiogenesis [35]. miR-210 appeared in both upstream regulators and causal networks in our analysis, and it upregulates VEGF expression, proliferation and migration of endothelial cells, and formation of sprouts during angiogenesis [36]. miR-210 is a hypoxia-induced miR, and its expression is reduced in DFUs. It has an inverse relation with HIF-1α and regulates hypoxic gene expression [37,38]. miR-210 attenuates keratinocyte proliferation and differentiation by targeting cell cycle proteins [39]. Narayana et al. reported that hyperglycemia decreases the hypoxia-induced increased expression of miR-210 in human and mouse diabetic wounds, and local administration of miR-210 accelerates wound healing in diabetic conditions [40]. miR-486 appeared in both upstream regulator and causal network analysis. miR-486-5p was found to be in a higher serum level in patients with metabolic syndrome [41] and extracellular vesicles with miR-486-5p promote wound healing by enhancing angiogenesis [42,43]. miR-324 appeared in both analyses, however, the literature does not document any role of miR-324 in diabetic wound healing. miR-324 attenuates wound healing assay (cell migration) in colorectal cells [44].

miR-186, miR-199, and miR-338 appeared in upstream regulator analysis while miR-155 and miR-1225 appeared in causal network analysis. miR-186-5p inhibits macrophage proliferation and migration during wound healing in mice by targeting TGF-βR2 and delays wound healing involving the TGFβR2/Smad2/p38 axis [45]. miR-199a-5p is significantly increased in DFU wound tissues, and its inhibition increases VEGF expression, significantly promoting wound healing [46]. miR-199a-3p is associated with scarring associated with burns and wound healing [47]. miR-199a-5p negatively regulates cell migration and angiogenesis during wound healing [28]. miR-338-3p is associated with increased synthetic VSMC phenotype [48] and inhibition of TNF-α-induced lipogenesis in human sebocytes [49]. However, its role in wound healing has not been investigated. miR-155 is involved in promoting DFU healing regulated by lncRNA CASC2, immune cell maturation, differentiation, function, and tissue regeneration affecting cells like fibroblasts [33]. miR-155 is pro-inflammatory during normal wound healing [50] and its inhibition decreases wound inflammation and increases FGF7 [51] and improves Th17/Th9 immune response [52] promoting wound healing [27]. miR-1225 is associated with attenuation of the p65 subunit of NF-κB in tumor biology [53], however, its role in wound healing warrants investigation.

In addition to the miRs appearing in upstream regulators and causal network analysis in IPA, network analysis of TF-miR interaction revealed miR-340, miR-142-3p, miR-377, miR-374a, miR-194, miR-891b, miR-506, miR-365, miR-600, miR-502-5p, miR-124, miR-20, miR-623, miR-495, miR-let 7a, and miR-let 7b (Figure 1 and Figure 2). Deficiency of miR-142, a family containing miR-142-3p and miR-142-5p expressed on immune cells, which is associated with neutrophil chemotaxis, increases the propensity of inflammation, contributing to delayed wound healing or non-healing of the wound [54]. miR-377 overexpression inhibits angiogenesis targeting CD133 and VEGF in the context of esophageal cancer [55]. miR-506-3p post-transcriptionally suppresses beclin-1 expression and regulates autophagy and proliferation of fibroblasts in the skin after burns [56]. An increased expression of miR-155 and miR-506-3p is associated with enhanced wound healing involving PI3K-Akt signaling in burns [57]. He et al. reported that exosomes containing MALAT1 enhance wound healing by targeting miR-123 via activation of the Wnt/β-catenin pathway [58]. miR-124-3p and miR-139-5p are involved in enhanced wound healing in porcine [59]. These results suggest that the miRs showing an interaction with TFs in this study may be a therapeutic target to promote wound healing [60,61]. However, the literature evidence is lacking for most of the miRs that appeared in our network analysis for their role in cutaneous wound healing and healing of DFUs, thus warranting investigations.

Since miRs involved in wound healing showed interaction with activated and inhibited TFs, it is important to understand the role of TFs in the regulation of wound healing, especially in the context of DFUs. The role of TFs AP1 (JUN/FOS), KLF5, and STAT3 in epidermal plasticity, SUZ12, EZH2, EED, NF-KB, KDMB6A, AP-1, and SMADs in initiation and early re-epithelialization, SMAD2, SMAD3, SMAD5, SMAD8, AP-1, TGF-β1, HIF-1α, EZH2, EGR-1, EED, and SUZ12 in keratinocyte migration during wound healing has been discussed [1,62]. NRF2 expression increases with increased oxidative stress due to ROS production after wounding. NRF regulates inflammation and keratinocyte and fibroblast proliferation and migration, playing a role in wound closure and re-epithelialization [9]. The SOX family of TFs, including SOX2, SOX7, and SOX9, play a critical role in promoting cell renewal, fibroblast phenotypic change, and keratinocyte proliferation and migration during wound healing [11]. The role of other TFs, including E2F-1, HOXA3, HOXD3, PPARα, PU.1 [63], Foxn1 [10], NR4A1 [17], FOXOs [12], high cambial activity2 (HCA2), target of monopteros6 (TMO6), DNA binding with one finger (DOF)2.1, DOF6 [21], and Sp1/Sp3 [64], in regulating molecular and cellular mechanisms during wound healing has been discussed. This study revealed TFs, including AP-1 (JUN/FOS), HIF1A, STAT3, EGR1, EZH2, and NFKB (RELA), as discussed above, activated in DFU tissues. Along with this, many additional TFs were also revealed in this study (Supplementary File S1) and by network analysis (Figure 3, Figure 4, Figure 5 and Figure 6).

**Figure 7:**
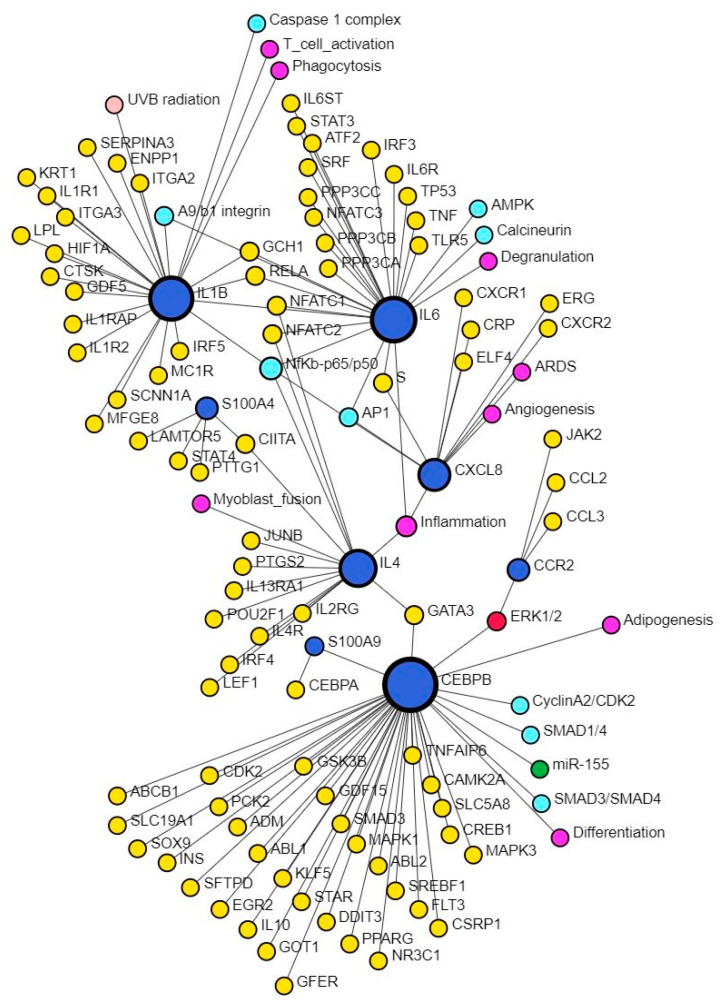
Network analysis revealing interaction between CXCL8 and S100 proteins. There was no direct interaction between CXCL8 and S100 proteins; however, there was an interaction of S100 proteins with CEBPB and IL-4, which in turn interacts with CXCL8 through NF-κB. This suggests that infiltration of immune cells and increased secretion of pro-inflammatory cytokines perpetuates inflammation, a major factor contributing to the non-healing of DFUs. S100 proteins are secreted by immune cells, which, on activation, secretes pro-inflammatory cytokines. Further, interaction of these proteins with inflammation, angiogenesis, differentiation, degranulation, phagocytosis, and T-cell activation suggests their role and the possibility of targeting them to promote DFU healing.

CXCL8, the gene encoding for IL-8, plays a critical role during wound healing by promoting angiogenesis in the early phase, but its persistently increased expression may contribute to chronic and non-healing wounds [3]. This study also revealed multiple TFs mostly activated except ARID1A, FOXA1, and BTG2 in upstream regulators and XBP1, NFKBIZ, ZNF366, HIVEP1, ZNF24, PITX1, and HIVEP1 (causal network analysis) which were inhibited (Supplementary File S1). S100A7, S100A8/A9, S100A12, and S100A15 play critical roles in tissue repair and regeneration, macrophage migration, invasion, and differentiation and regulation of inflammation [65], which are the molecular mechanisms playing a critical role in wound healing. Increased expression of S100A8 and IL-8 promotes persistent inflammation and alters wound healing [66], however, Su et al. reported that treatment of wounds with S100A8-overexpressing adipose-derived stem cells promotes wound healing [67]. The interaction between CXCL8 and S100 proteins was evident in network analysis using SIGNOR data (Figure 7). Our study revealed EHF, TP53, and NFKBIZ (activated), and KMT2D (inhibited) in upstream regulators analysis while MAZ, NONO, ZIC5, TP63, EHF, and NFKBIZ (activated), KMT2D, NFKBIZ, and ZNF366 (inhibited) were revealed in causal network analysis regulating S100 proteins in DFU tissues compared to control tissues.

ARID1A was activated in DFU tissues in this study, and increased expression of ARID1A is associated with delayed wound healing, while knocking out ARID1A improves wound healing [68,69]. The definitive role of CEBPA in wound healing is not well established; however, it is involved in miR-TF interaction regulating wound healing [61]. CEBPB was activated in DFU tissues, and its role in wound healing in DFUs is not known. CXCL8 and S100A9 were, among others, target molecules of CEBPB, and this suggests that activation of CEBPB will increase CXCL8 expression, contributing to persistent inflammation and non-healing DFUs. ESCIT regulates the ECSIT signaling integrator gene, enabling DNA-binding transcription factor activity and chromatin-binding activity to play a role in BMP signaling pathways. Evolutionarily conserved signaling intermediate in Toll pathways (ECSIT) protein is involved in the activation of NF-κB and ROS production in mitochondria [70]; however, its role in DFU healing is unknown. MED1 (Mediator complex subunit 1), a co-activator for multiple TFs, was activated in DFU tissues and regulates keratinocyte proliferation during wound healing [71], and MED1 null mice developed epidermal hyperplasia; however, Meng et al. reported that ablation of MED1 in oral keratinocytes promotes oral mucosal healing involving JNK signaling [72].

NFKBIZ, activated in DFU tissues, regulates the expression of NF-κB and plays a critical role in wound healing by interacting with other TFs [73,74]. STAT3 and SP4 are involved in increased angiogenic endothelial tube formation when human peritoneal mesothelial cells are treated with IL-6 and serum IL-6R together [75]. Since SP4 is involved in increased angiogenesis, SP4 should promote wound healing but activated SP4 in chronic non-healing DFUs contradicts the notion and must be investigated. SPI1 was activated in DFU samples, but its role in wound healing is not well understood; however, network analysis by Arodz et al. [61] revealed SPI1 activated in immune cells during wound healing. TP53 was activated in non-healing DFU tissues in this study, and increased TP53 may be the reason for non-healing because silencing of p53 is associated with increased expression of vasculogenic cytokines and endothelial cell markers in association with enhanced wound healing [76]. In addition to the factors activated in DFU tissues and described in this section, the role of other TFs, including CEBPB, CEBPA, ECSIT, EHF, ELF4, EP300, FOXL2, MAZ, NFE2L2, PPRC1, ZHX2, and ZIC5 found activated in non-healing DFU tissues is unknown. However, the interaction of these TFs with other TFs, including HIF1A, EGR1, NFKBIZ, SPI1, JUNB, RELA, SP4, and others playing a crucial role in diabetic wound healing (Figure 3, Figure 4, Figure 5 and Figure 6) suggest their role in DFU healing. Investigating the role of these TFs in DFU healing is rationalized by the fact that these TFs regulate various genes involved in DFU healing (Supplementary File S1) like CEBPB targets CXCL8 [3,77], STAT3 [78], CCL20 [79], IRS1 [80], and MMP10 [81] playing a regulatory role in DFU or wound healing. Similarly, CEBPA targets CXCL8, IL1, S100A9; ECSIT targets CCL3L1, CXCL8, and NFKB1; molecular targets of EHF are CCL20, S100A7, S100A8, S100A12, and VNN1, ELF4, FOXL2, and NEF2L2 targeting CCL20; PPRC1 targeting CCL20 and CXCL8; ZHX2 targeting HIF1A; and MAZ targeting CCL20, CXCL8, various keratins and MMPs.

**Figure 8:**
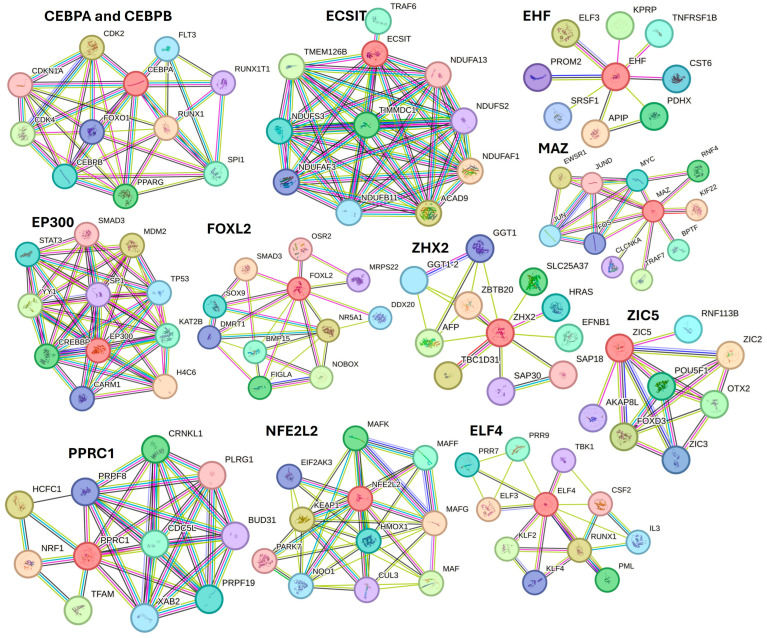
STRING network for activated transcription factors CEBPB, CEBPA, ECSIT, EHF, E74 like ETS transcription factor 4 (ELF4), EP300, FOXL2, MAZ, NFE2L2, PPRC1, ZHX2, and ZIC5.

Further, STRING network analysis predicting protein–protein interactions (Figure 8) for proteins regulated by activated TFs showed their interaction with various factors involved in DFU healing, wound healing, or diabetes (as discussed in the text).

For example, protein CEBPA and CEBPB showed interaction with FOXO1, PPARG, and SPI1; ECSIT with TRAF6 (a downstrem effector of TLR4); EHF with TNFRSF1B; EP300 with STAT3, SMAD3, TP53, SP1, and YY1; FOXL2 with SMAD3, SOX9, and BMP16; ZHX2 with AFP; MAZ with JUN and FOS (AP1); PPRC1 with NRF1; NFE2L2 with HMOX1 and KEAP1; ELF4 with TBK1, CSF2, RUNX1, and KLF4. These interactions suggest that these TFs may play a critical role in DFU healing.

**Figure 9:**
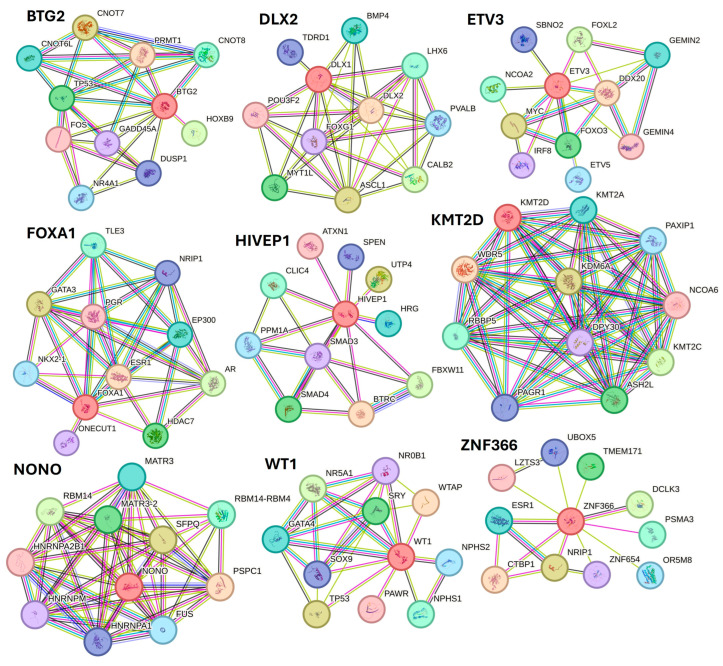
STRING network for transcription factors BTG2, DLX1, ETV3, FOXA1, HIVEP1, KMT2D, NONO, WT1, and ZNF366.

Along with various activated TFs, the study results also revealed various TFs, including BTG2, DLX1, ETV3, FOXA1, HIVEP1, KMT2D, NANOG, NONO, PITX1, WT1, XBP1, ZNF24, and ZNF366 inhibited in non-healing DFU tissues. Xiang et al. [82] reported downregulation of NANOG in DFU healers, but in our study, NANONG expression was inhibited in non-healers. PITX1 primes human oral wounds for healing [83], and a decreased expression in non-healing DFU suggests that a decreased expression of PITX1 may be the cause of the non-healing nature. XBP1 was inhibited in non-healing DFUs in this study, and inhibition of XBP1 may be the reason for non-healing because wound healing is accelerated by boosting unfolded protein response (UPR) transcriptional activator XBP1 [84]. The role of linc00174-EZH2-ZNF24/Runx1-VEGFA in epigenetically regulating wound healing after burn has been reported [85]; however, its role in DFU healing is unknown and should be investigated. The role of inhibited TFs namely BTG2, DLX1, ETV3, FOXA1, HIVEP1, KMT2D, NONO, WT1, and ZNF366 in DFU wound healing is unknown and warrant investigation. STRING network analysis, predicting protein-protein interactions for proteins regulated by inhibited TFs showed their interaction with various factors involved in DFU healing, wound healing, or diabetes (Figure 9).

STRING network showed interaction of protein BTG2 with TP53 and FOS; DLX2 with BMP4 and POU3F2; ETV3 with FOXL2, MYC, and FOXO3; FOXA1 with FOXA1, EP300, and ESR1; HIVEP1 with SMAD3 and SMAD4; KMT2D with WDR5 and NCPA6; NONO with RBM14; WT1 with TP53 and SOX9; and ZNF366 with ESR1, CTBP1, and NRIP1 among others. The interaction between these proteins (protein complexity) suggests that these TFs may play a critical role in DFU healing.

Delineating the role of the activated and inhibited TFs interacting with miR and TFs known to have a role in DFU healing should be investigated because of their interactions (Figure 3, Figure 4, Figure 5 and Figure 6). This notion is supported by the fact that these TFs target various genes/molecules (Supplementary File S1) playing a regulatory role in DFU healing such as BTG2 targets CCL20, CXCL8, and IL1; FOXA1 targeting CXCL8; HIVEP1 targeting IL1A, IRAK2, MAPK, and NFKB; KMT2D and XBP1 targeting keratins, interleukins, cytokines, S100 proteins, and SMADs; and PITX1 targeting CXCL8, NFKB, and interleukins. It is also evident from Supplementary File S1 that a single molecular target is regulated by multiple TFs, and this suggests the existence of a complex proteome in the DFU environment, which must be investigated. This complex proteome also be due to the presence of different microbes or dysbiosis and warrants investigation.

Investigating the effects of activation and inhibition of TFs and miRs in chronic nonhealing ulcers is important because this will lead to altered genes and, thereby, protein expression. Altered protein expression contributes to non-healing in DFUs, and proteomic analysis revealed that MMP9, fatty acid-binding protein 5 (FABP5), and integrin subunit alpha M (ITGAM) are potential targets [86]. Negative-pressure wound therapy (NPWT) promotes wound healing, and Jia et al. reported a change in the protein expression after NPWT with a significantly higher expression of protein S isoform 1, inter α-trypsin inhibitor heavy chain H4, and peroxiredoxin-2 and significantly lower expression of cathepsin S [87]. Another study reported upregulation of proteins LRG1, CD5L, CRP, IGHA1, and LBP in DFU [88]. These results suggest that changing protein expression is associated with pathogenesis as well as non-healing of DFUs. Altered gene expression, post-translational modification of proteins, different proteoforms, and protein species, and complex interaction between proteins (proteome complexity), which changes with every phase of healing, may contribute to the non-healing of DFUs. Thus, conducting proteomics on healthy and DFU tissues, identifying different proteoforms, protein species, the effects of different protein diets, and the complex interaction between different proteins/combinations of proteins may help in designing improved therapeutics to improve clinical outcomes. Activated and inhibited TFs and miRs found in this study, which may alter protein expression and target proteins favoring DFU healing, warrant further research.

## 5. Conclusions

The results of this study revealed many activated and inhibited transcription factors and microRNA associated with non-healing chronic DFUs in humans. These TFs and miRs are associated with multiple factors playing a critical role in wound healing, diabetes, and DFU healing. The factors regulated by activated and inhibited TFs and miRs are involved in regulating fibroblasts, keratinocytes, VSMCs, and endothelial cell proliferation and migration, inflammation, and re-epithelialization. Regulation of CXCL8, CCL20, and S100 proteins, among other targets differentially expressed in DFUs, warrants investigations and may serve as therapeutic targets. Further, only a few studies are conducting proteomic analysis of DFUs and investigating the changing protein expression; there is a need for more research to delineate the changing protein expression during DFU pathogenesis, responsible for non-healing and differentially expressed in chronic non-healing ulcers compared to healing ulcers. Analyzing the transcriptomics data in combination with proteomics data will reveal the interactions of TFs and miRs associated with changing protein expression, and targeting them may have therapeutic advantages.

## Figures and Tables

**Table 1:** Activated and inhibited transcription factors expressed in diabetic foot ulcer tissues compared to non-ulcerated tissues in upstream and causal network analysis.

Upstream Regulator Analysis			Causal Network Analysis		
Transcriptional Regulators	Activation Status	Activation z-Score	Transcriptional Regulators	Activation Status	Activation z-Score
EHF	Activated	2.714	MAZ	Activated	6.042
NONO	Activated	3.984	NONO	Activated	3.77
TP63	Activated	4.615	ZIC5	Activated	4.346
NFKBIZ	Activated	2.755	TP63	Activated	4.743
RELA	Activated	3.49	EHF	Activated	3.683
PPRC1	Activated	3.064	EHF	Activated	2.683
FOXL2	Activated	2.811	RELA	Activated	4.111
FOS	Activated	2.732	NONO	Activated	4.082
ECSIT	Activated	2.63	ECSIT	Activated	4.004
JUNB	Activated	2.236	NFKBIZ	Activated	2.828
CEBPA	Activated	3.208	PPRC1	Activated	3.051
HIF1A	Activated	2.431	RELA	Activated	3.545
EZH2	Activated	2.668	FOXL2	Activated	2.887
CEBPB	Activated	2.148	FOS	Activated	2.985
STAT3	Activated	2.636	FOS	Activated	2.887
SPI1	Activated	2.954	FOS	Activated	2.714
EP300	Activated	2.415	ECSIT	Activated	2.646
ELF4	Activated	2	SP4	Activated	2.236
EGR1	Activated	2.38	ZHX2	Activated	2
NFE2L2	Activated	2.236	XBP1	Inhibited	−2.776
MED1	Activated	2.449	KMT2D	Inhibited	−5.303
KMT2D	Inhibited	−3.444	NFKBIZ	Inhibited	−2.944
ARID1A	Inhibited	−2.626	ZNF366	Inhibited	−3.28
HIVEP1	Inhibited	−2.53	KMT2D	Inhibited	−3.5
NANOG	Inhibited	−2.138	HIVEP1	Inhibited	−3.772
FOXA1	Inhibited	−2.101	ZNF24	Inhibited	−3.674
DLX1	Inhibited	−2.121	PITX1	Inhibited	−2.183
ETV3	Inhibited	−2.646	HIVEP1	Inhibited	−2.53
BTG2	Inhibited	−2			
WT1	Inhibited	−2.219			

## Data Availability

All data related to this manuscript are included in the manuscript and as a Supplementary File. The DEG profile data has been submitted to the public database at https://submit.ncbi.nlm.nih.gov/subs/sra/SUB14735487/ (accessed on 17 September 2024).

## Supplementary Materials

The following supporting information can be downloaded at: https://www.mdpi.com/article/10.3390/proteomes12040032/s1, File S1: Molecular Targets of Activated and Inhibited Transcription Factors and microRNAs.
